# Supplementary material for: Eps15 Homology Domain-Containing Protein 3 Hypermethylation as a Prognostic and Predictive Marker for Colorectal Cancer
Source: Biomedicines. 2021 Apr 22;9(5):453. doi: 10.3390/biomedicines9050453 (PMC8145505; doi:10.3390/biomedicines9050453)
Supplement: Supplementary file 1 [file biomedicines-09-00453-s001.zip › biomedicines-1161795-SI.pdf]

biomedicines

MDPI

Article

Eps15 Homology Domain-Containing Protein 3 Hypermethylation as a Prognostic and Predictive Marker for Colorectal Cancer

SUPPLEMENTARY MATERIALS

Table S1. Clinical parameters of 26 paired patients with CRC whose tissues were analyzed with Illumina Infinium HumanMethylation450 BeadChip array assays.

Characteristics	Total *n*(%)
**Total**	26
**Clinicopathological parameters**	
**Age**	
< 60	8 (30.8)
> 60	18 (69.2)
**Gender**	
Male	18 (69.2)
Female	8 (30.8)
**Tumor type**	
Adeno	21 (84.0)
Mucinous	1 (4.0)
Others	3 (12.0)
**Tumor stage**	
Early (stage I and II)	16 (61.5)
Late (stage III and IV)	10 (38.5)
**Primary tumor**	
Submucosa	1 (3.8)
Muscularis propria	5 (19.2)
Subserosa	17 (65.4)
Penetrate the visceral	3 (11.5)
**Regional lymph nodes**	
No regional lymph node metastasis	17 (65.4)
Metastasis in regional lymph nodes	9 (34.6)
**Distant metastasis**	
No distant metastasis	24 (92.3)
Distant metastasis	2 (7.7)
**Differentiation grade**	
Well	3 (11.5)
Moderate	18 (69.2)
Poor	5 (19.2)
**Location**	
Colon	20 (76.9)
Rectal	6 (23.1)

Table S2. Cox proportional-hazards survival analysis in Western patients with CRC.

Variable.	Multivariate Analysis[a]		
	**HR**	**95%CI**	***p*-value**
**Overall Survival**			
Age	1.140	0.650 – 2.000	0.649
Location	1.324	0.694 – 2.527	0.395

Tumor size	2.607	0.785 – 8.655	0.118
Metastasis	1.000	0.478 – 2.092	1.000
Lymph invasion	2.396	1.311 – 4.279	0.005**
EHD3 DNA methylation	1.963	1.036 – 3.719	0.039*
EHD3 RNA expression	1.579	0.863 – 2.890	0.138
**Recurrence-free survival**			
Age	1.037	0.475 – 2.264	0.927
Location	1.737	0.680 – 4.438	0.248
Tumor size	2.008	0.448 – 8.999	0.362
Metastasis	1.137	0.448 – 2.899	0.787
Lymph invasion	3.485	1.552 – 7.821	0.002**
EHD3 DNA methylation	2.180	0.191 – 1.033	0.059
EHD3 RNA expression	0.444	0.923 – 5.152	0.076
**Progression-free survival**			
Age	0.889	0.246 – 3.213	0.858
Location	7.514	0.736 – 76.725	0.089
Tumor size	0.448	0.074 – 2.703	0.381
Metastasis	16.494	2.149 – 126.620	0.007**
Lymph invasion	0.451	0.050 – 4.059	0.477
EHD3 DNA methylation	6.026	0.666 – 54.489	0.110
EHD3 RNA expression	0.287	0.058 – 1.420	0.126

*$p < 0.05$; **$p < 0.01$; ***$p < 0.001$.

a. For multivariate Cox proportional-hazards survival analysis, the data were adjusted for age, differentiation, sex, tumor stage, and location.
b. *EHD3* methylation levels were derived from CRC tumors in the TCGA dataset.

Table S3. List of primer sequences and reaction conditions used in the study.

Real-Time RT-PCR		Sequence (5' to 3')	Probe	Tm (°C)
EHD3	Forward	GGGACTTCCCCAATCTGAAG	#67	60
	Reverse	GCCAGCATGTCGTCCACT		
GAPDH	Forward	AGCCACATCGCTCAGACAC	#60	60
	Reverse	GCCCAATACGACCAAATCC		
		QMSP		
EHD3	Forward	CGTCGTCGTTTTTTTTGAGTC		60
	Reverse	TACAAAAAAACTACCAAATACCGAA		
	probe	TACTTCGACTCCGCTCCTACCGAACC		
ACTB	Forward	TGGTGATGGAGGAGGTTTAGTAAGT		60
	Reverse	AACCAATAAAACCTACTCCTCCCTTAA		
	probe	ACCACCACCCAACACACAATAACAAACACA		

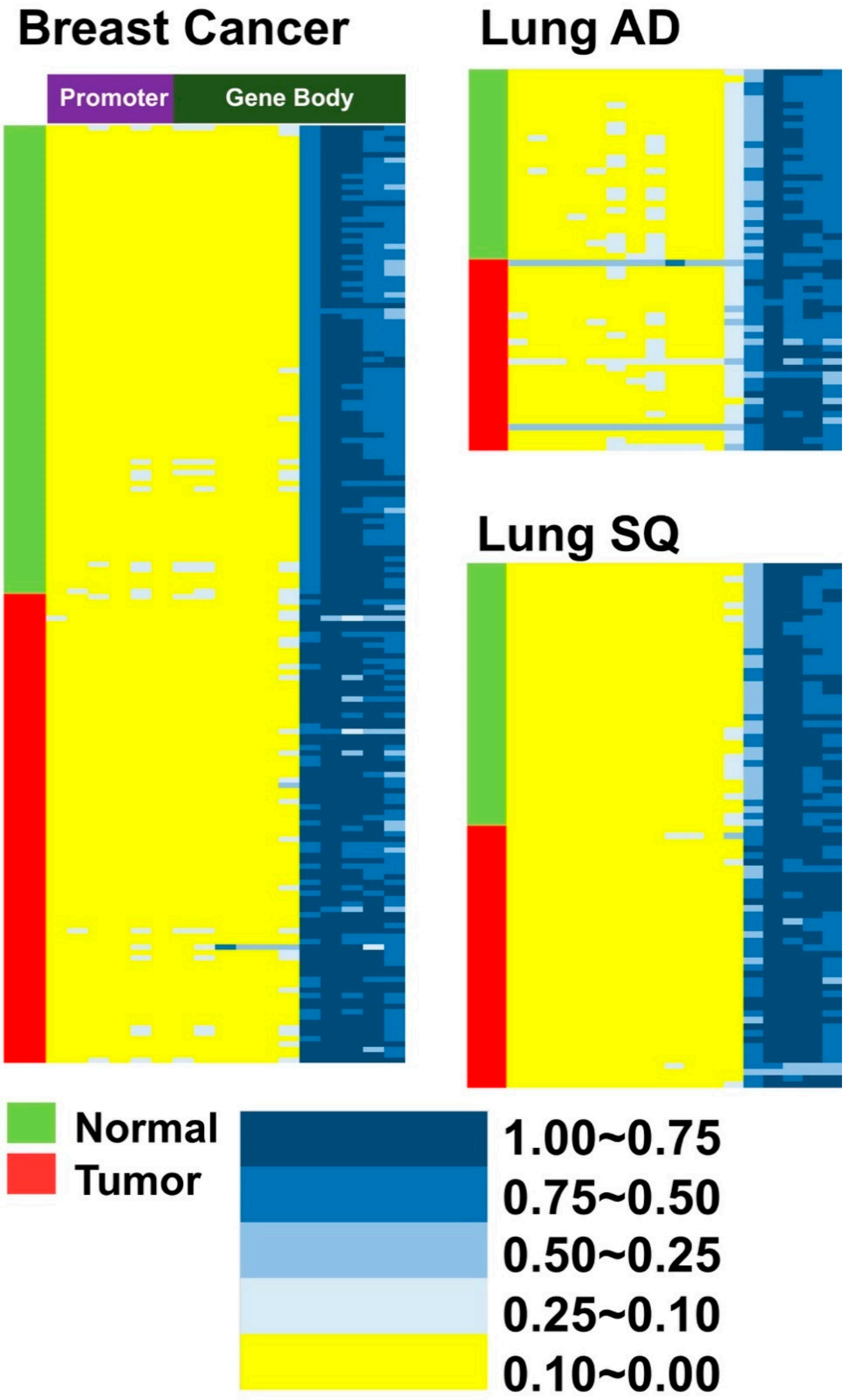

Figure S1. Methylation heatmap of *EHD3* in paired breast cancer, lung adenocarcinoma, and squamous cell lung cancer tissues.

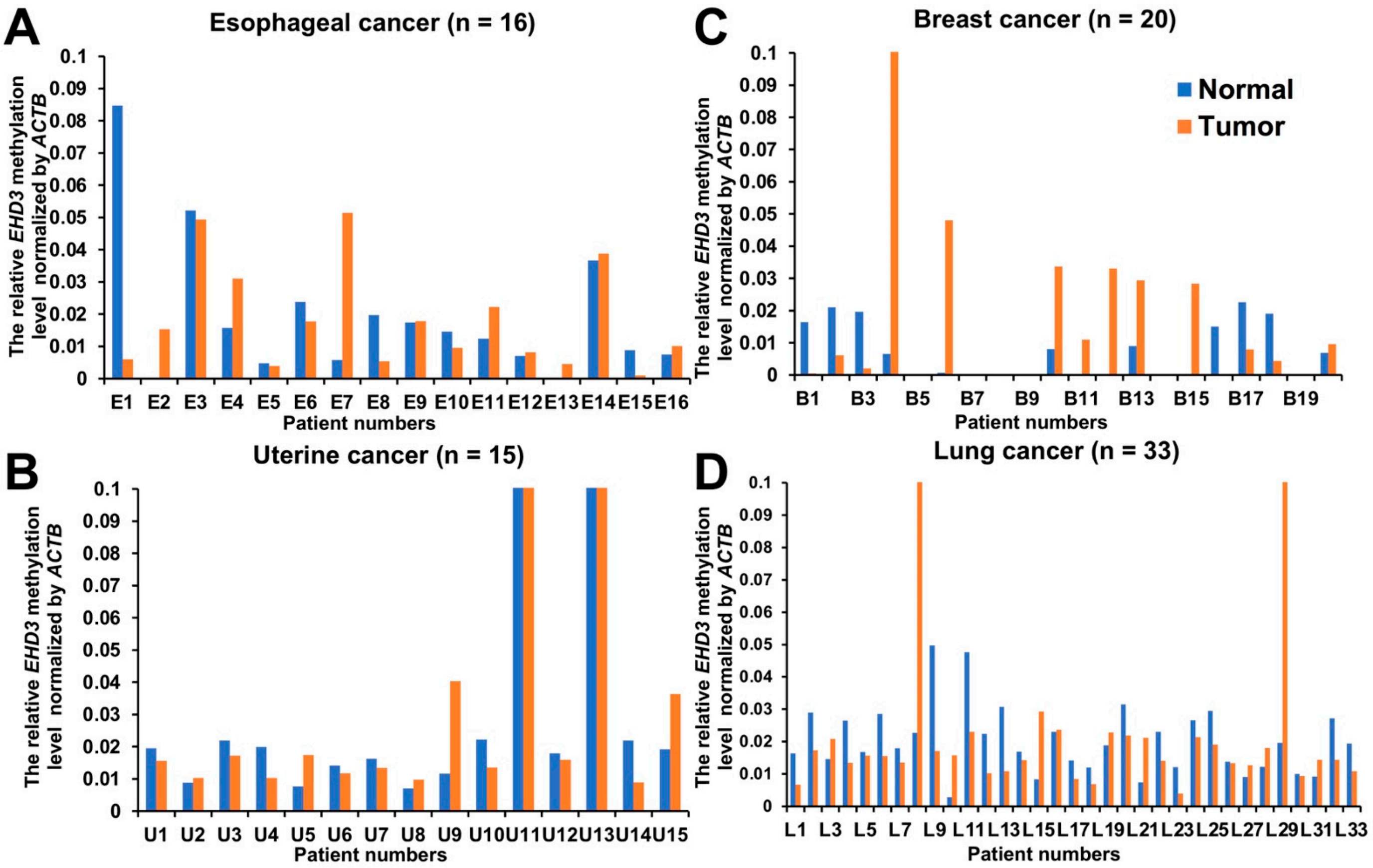

Figure S2. Bar graph of the promoter hypermethylation of the *EHD3* pattern in paired (**A**) esophageal, (**B**) uterine, (**C**) breast, and (**D**) lung cancer tissues compared with matched normal tissue in the Taiwanese cohort.

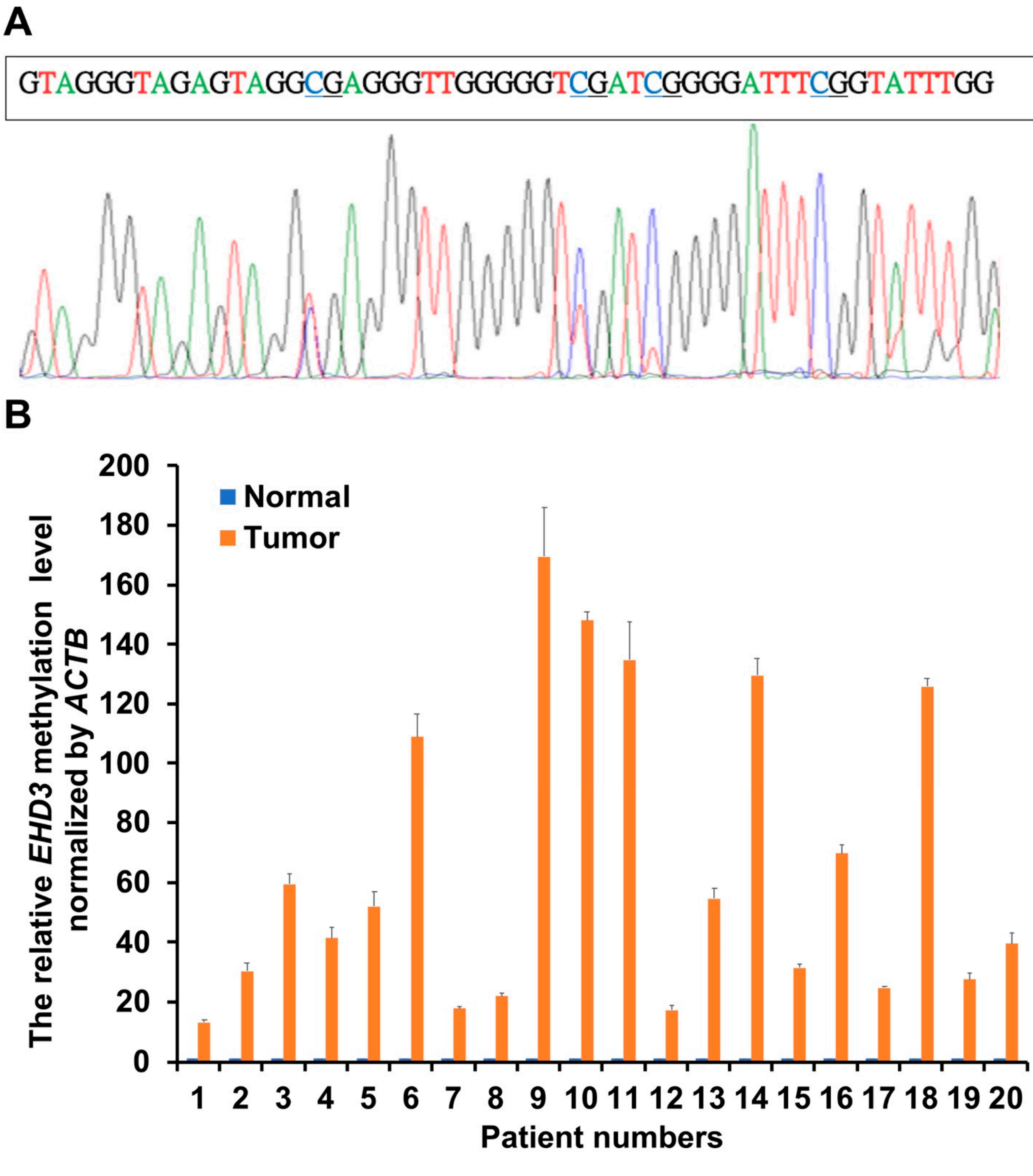

Figure S3. Hypermethylation of the *EHD3* promoter gene was analyzed in patients with CRC. (**A**) Bisulfite sequencing was performed to verify the specificity of the *EHD3* QMSP assay. (**B**) Representative figure of *EHD3* methylation determined through QMSP assay in 20 patients with CRC. Data are presented as means ± standard deviations. Experiments were performed with at least three technical replicates.

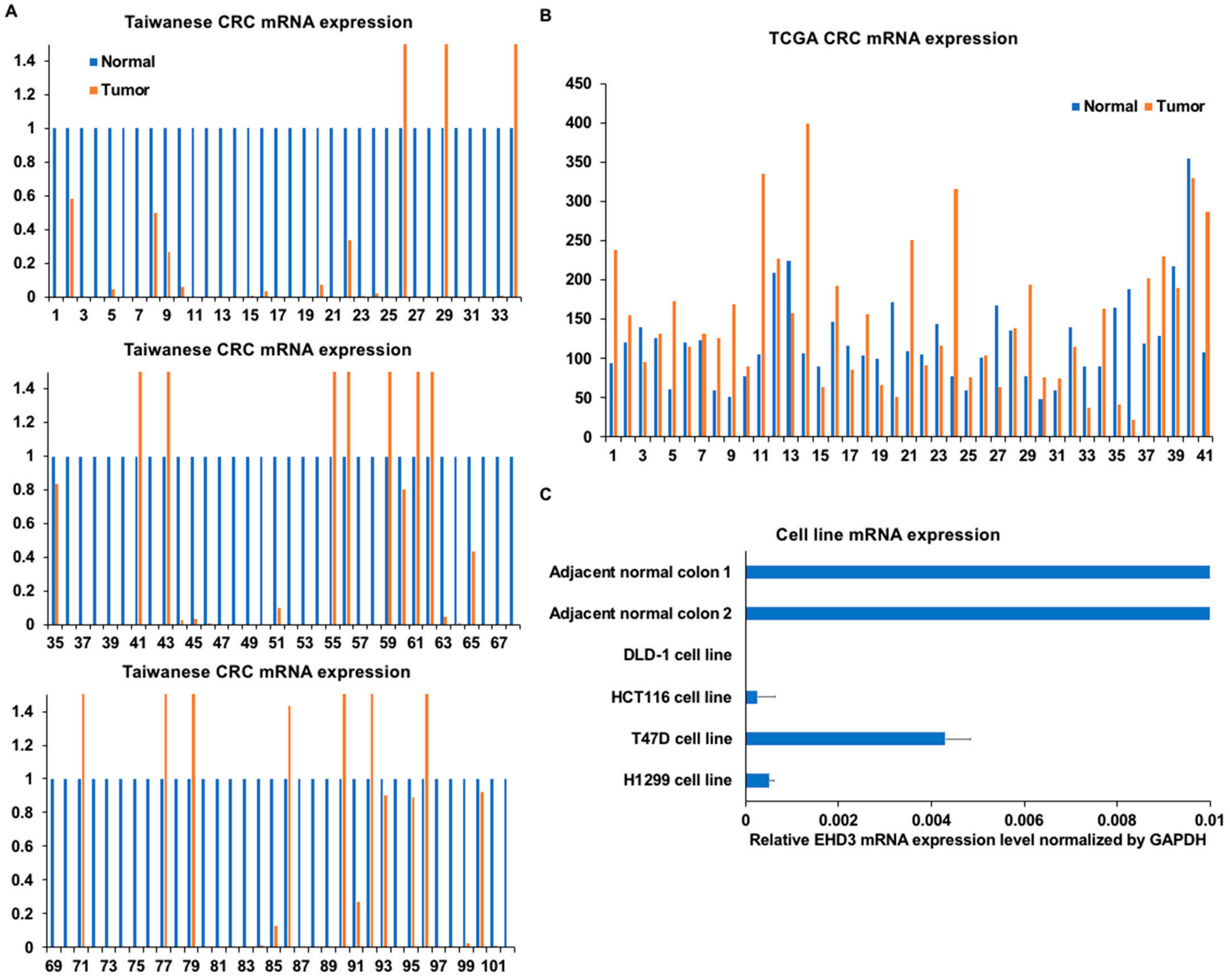

Figure S4. *EHD3* mRNA expression levels were analyzed in CRC tumors and paired normal colorectal tissues. (**A**) *EHD3* mRNA expression levels were analyzed using real-time RT-PCR in 102 Taiwanese patients. (**B**) *EHD3* mRNA expression levels were analyzed with the RNA sequencing of paired tissues from normal controls and 41 paired patients with CRC from the TCGA.(**C**) *EHD3* mRNA expression levels were analyzed in two adjacent normal colon tissues, two colon cancer cell lines (DLD-1 and HCT116), one breast cancer cell line (T47D), and one lung cancer cell line (H1299).
